# Different culture method changing CD105 expression in amniotic fluid MSCs without affecting differentiation ability or immune function

**DOI:** 10.1111/jcmm.15081

**Published:** 2020-03-02

**Authors:** Ding Wang, Nengqing Liu, Yingjun Xie, Bing Song, Shu Kong, Xiaofang Sun

**Affiliations:** ^1^ Key Laboratory for Major Obstetric Diseases of Guangdong Province Key Laboratory of Reproduction and Genetics of Guangdong Higher Education Institutes Experimental Department of Institute of Gynecology and Obstetrics The Third Affiliated Hospital of Guangzhou Medical University Guangzhou China

**Keywords:** amniotic fluid MSCs, CD105, cell culture, differentiation ability, immune function

## Abstract

MSCs are kind of cultured cells that reside in different tissues as inducers or regulators of physiological and pathological processes. Here, we derived MSCs from amniotic fluid and compared their differentiation ability and immunosuppression effect on PHA‐activated PBMC with those of MSCs isolated from umbilical cords. Amniotic fluid MSCs were isolated and cultured on commercial AFC medium and classic MSC medium, and the number and size of colonies were used to evaluate differences in primary and passaged culture. Rate of proliferation, population doubling time, cell morphology, cell surface markers and mRNA expression were measured in subcultured cells. Furthermore, a comparative study was performed with umbilical cord MSCs to assess the ability of differentiation and immunosuppressive effect of PHA‐stimulated PBMCs. Amniotic fluid MSCs were isolated and expanded by three methods, and exhibited nearly all the characteristics of umbilical cord MSCs. Compared with umbilical cord MSCs, amniotic fluid MSCs had an enhanced osteogenic and chrondrogenic differentiation capability, and stronger immunosuppression effect of inhibition of PHA‐activated PBMC division. Culture with commercial AFCs medium yielded the highest percentage of CD105 expression and showed some advantages in primary cell isolation, cell source‐specific marker retention and cell proliferation. We demonstrated that amniotic fluid MSCs exhibited some advantages over umbilical cord MSCs, and different culture media caused cell proliferation, cell surface marker and cell morphology change, but were not associated with varying differentiation capability and immune effects.

## 1 INTRODUCTION

MSCs are popular for cytotherapy, and there are at least five main names: mesenchymal stem cells,^1^ marrow stromal cells,^2^ multipotential mesenchymal stromal cells,^3^ mesenchymal stromal cells^4^ and medicinal signalling cells.^5^ Different terms and invariant acronym (MSCs) were resulting some unproven cytotherapys were sold,^6^ but there were same criteria for definition of MSCs.^3^ MSCs are somatic stem cells that have characteristic self‐renewal and multipotent differentiation abilities. In vitro, MSCs could be differentiated into cells from different germ layers by specific inducers. The most reasonable application of MSCs regeneration potential is in the promotion of bone regeneration and the maintenance of haematopoietic stem cells. Alternative descriptions of MSCs are due to their biological functions not as stem cells but as modulators of the immune system or as beneficial agents in different disease processes, which was the key reason for their use in clinical medical care. According to registered trials,^7^ thousands of MSC projects for human disease care are in progress. MSCs can be derived from multiple tissues, including bone marrow (BMMSCs), umbilical cords (UCMSCs) and amniotic fluid.^8^ MSCs are non‐immortalized, adherent, spindle‐like cells,^9^ and their primary culture for expansion is performed in basal medium and foetal bovine serum in most studies.^10^ Furthermore, in order to produce animal origin free human MSCs, chemical defined media,^11^ cord blood serum^12^ and human blood derivatives^13^ was used to replace FBS. The promotion of culture media and methods will be of benefit to MSCs basic study and clinical application.

Amniotic fluid is the environment in which a foetus grows, and it is composed of the excretory products of the foetus (urine is the main component) and accompanies the foetus throughout the whole pregnancy. Types of amniotic fluid cells (AFCs) are changing in different terms of pregnancy according to foetus development. For the first trimester, there are few AFCs that share features of embryonic stem cells (ESCs), including cell morphology, molecular markers and the ability to differentiate into the three germ linages in vitro (form embryoid bodies) and in vivo (form teratomas).^14^ It is easy to isolate and culture AFCs for prenatal genetic diagnosis because in the second and early third trimesters they attach to plastic; their characteristics share a lot with MSCs, which already exhibit great potential in regenerative medicine applications.^15^, ^16^ Cells from term amniotic fluid are also regarded as potential cell therapy agents.^17^

In the current study, we established medium influence on isolation and culture of human amniotic fluid mesenchymal stem cells (AFMSCs) from the amniotic fluid during the second trimester. AFMSCs were expanded in commercial AFC medium and classic MSC medium in primary and passaging. Then, different cells, including expanded AFMSCs from different culture methods, UCMSCs and foreskin fibroblast cells, were subjected to systematic comparisons of the following parameters: mesenchymal specific cell surface markers, pluripotency marker mRNA levels, differentiation capability into mesenchymal cell linages and the immunosuppression effect of phytohemagglutinin^18^‐stimulated peripheral blood mononuclear cells (PBMCs). Our work will be beneficial for AFMSC‐associated cell biology and potential MSC therapeutic use.

## 2 MATERIALS AND METHODS

### 2.1 Cell culture

**Figure 1:**
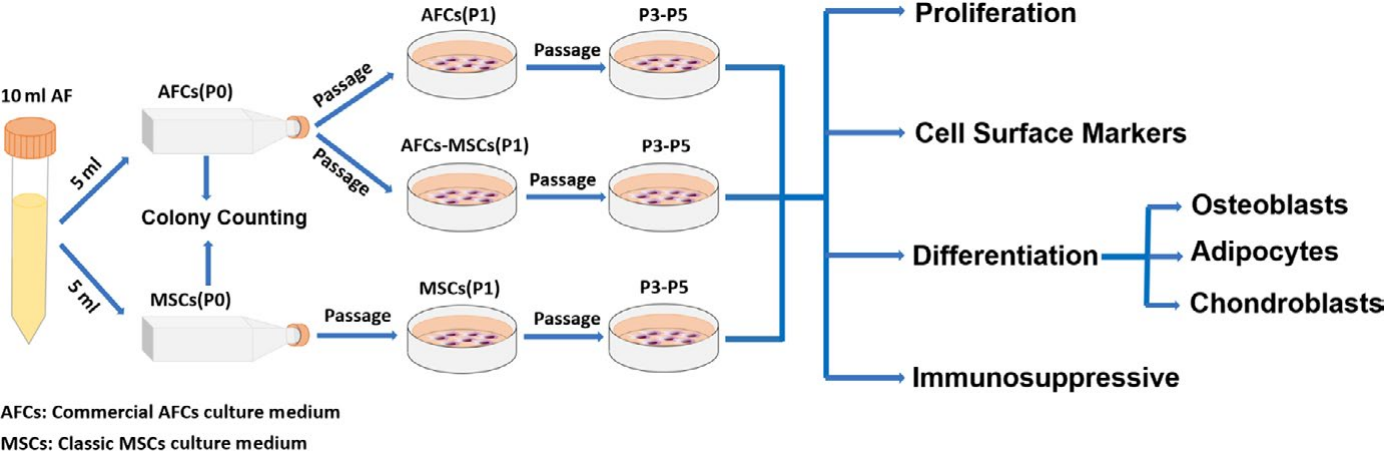
Schematic representation of AFMSCs isolation, culture and identification

This study was approved by the Ethics Committee of the Third Affiliated Hospital of Guangzhou Medical University, and all clinical samples were taken after informing the patients of the study and receiving signed consent. Ten millilitres of amniotic fluid from each patient was used in this study. The pregnant women who participated in this study were at the gestational age of 16‐20 weeks and suggested for cytogenetic prenatal diagnosis as high risk of serological screening and presentation of normal karyotypes. AFCs were cultured in two commercial AFC culture medium and classic MSC culture medium. The two commercial AFC culture medium were marked AFC1 (Baiyunshan, Cat# 20190502) and AFC2 (BI, Biological Industries Israel Beit Haemek LTD., Cat# 01‐194‐1BCS). The classic MSC culture medium was Dulbecco's modified Eagle's medium (CORNING, Cat# 10014CVR) supplemented with 10% foetal bovine serum (FBS) (Gibco, Thermo Fisher Scientific, Cat# 10099141), 100 U/mL penicillin and 100 μg/mL streptomycin (Gibco, Cat# 15140122). The culture process is shown in a schematic figure (Figure 1). Isolated UCMSCs were from tissue cultures of Wharton's jelly, which was isolated from umbilical cords, and fibroblasts were derived from foreskin. UCMSCs and fibroblasts were cultured in classic MSC culture medium at 37°C and 5% CO2 and were passaged as AFMSCs were, with trypsin‐EDTA (Gibco, Cat# 25300054).

### 2.2 Colony formation assay

We used primary amniotic fluid and passaged single AFCs to assay colony formation ability of different medium. Cell pellets from 1 mL, 5 mL of amniotic fluid and 100 passaged AFCs were seeded into 10 cm^2^ dishes, and crystal purple staining (Solarbio, Cat# G1062) was performed on the 9th day of primary culture. The number of colonies and the diameter of each culture were used for statistical analysis.

### 2.3 Cell viability assay

The MSCs were seeded in triplicated at a density of 5000 cells/well of a 96‐well plate. Cell proliferation and cell attachment were determined by cell viability, which was assessed by a commercial Cell Counting Kit‐8 (CCK‐8, Dojindo Molecular Technologies, Cat# CK04), according to the manufacturer's instructions.

### 2.4 Cell PD time assay

Cell PD time in relation to AFMSCs was calculated from passages 1‐6. Cells were stained with trypan blue (Invitrogen, Thermo Fisher Scientific, Cat# T10282) to identify living cells, and they were then counted with a cell counter (Countess™ II Automated Cell Counter, AMQAX1000, Thermo Fisher Scientific). For each passage, there were 1 × 10^5^ cells seeded in a 10 cm^2^ dish, and cells were removed for counting when at approximately 80%‐90% confluence. PD time was calculated according to the final cell number according to an online tool (http://www.doubling-time.com/Compute.php).

### 2.5 Flow cytometry analysis

Surface markers of MSCs were assayed by flow cytometry analysis (Attune™ NxT Acoustic Focusing Cytometer, A24863, Thermo Fisher Scientific), and the same voltage parameters for FSC and SSC were used in all the assays; FSC was 90, and SSC was 255. Fluorescently labelled antibodies were CD147‐FITC (Biolegend, Cat# 306204), CD90‐PerCP/Cyanine5.5 (Biolegend, Cat# 328117), CD44‐PE/CY7 (Biolegend, Cat# 103029), CD34‐FITC (BD Biosciences, Cat# 560942), CD73‐APC (Biolegend, Cat# 344005), CD105‐PE (Biolegend, Cat# 800503), CD14‐FITC (Biolegend, Cat# 301803), CD45‐APC (BD Biosciences, Cat# 555485), CD29‐PE (Biolegend, Cat# 303003), PCNA‐PE (Biolegend, Cat# 307908) and Ki‐67‐APC (Biolegend, Cat# 350513).

### 2.6 Quantitative real‐time polymerase chain reaction (RT‐PCR) analysis

Total RNA was extracted using TRIzol™ (Invitrogen, Cat# 12183555). cDNA was synthesized by PrimeScript™RT reagent Kit (Takara Biotechnology, Cat# RR047A). Quantitative polymerase chain reactions were conducted on a StepOne™ Real‐Time PCR System (Thermo Fisher Scientific, Cat#4376373) with SYBR^®^ Premix Ex Taq™ II(Takara Biotechnology, Cat# RR820A) and analysed using ViiA7™ System software (Thermo Fisher Scientific). The primers used are listed in Table 1. GAPDH was used as a control.

**Table 1:** RT‐PCR primers

Genes	Primers (5′‐3′)	
	Forward	Reverse
SOX2	GCTACAGCATGATGCAGGACCA	TCTGCGAGCTGGTCATGGAGTT
NANOG	CTCCAACATCCTGAACCTCAGC	CGTCACACCATTGCTATTCTTCG
OCT4	CCTGAAGCAGAAGAGGATCACC	AAAGCGGCAGATGGTCGTTTGG
CDKN2A	CTCGTGCTGATGCTACTGAGGA	GGTCGGCGCAGTTGGGCTCC
KSP	AGCCTATCCACCTGGCAGAGAA	TCTGGTCACGTAGAGGTTTCCC
SFTPA1	CACCTGGAGAAATGCCATGTCC	AAGTCGTGGAGTGTGGCTTGGA
NKX2.1	CAGGACACCATGAGGAACAGCG	GCCATGTTCTTGCTCACGTCCC
KRT2	ACCTACCGCAAACTGCTGGAGG	CAGAACCTCCAAAGGCAGCCTT
OST	CGAGGTGATAGTGTGGTTTATGG	GCACCATTCAACTCCTCGCTTTC
ALP	GCTGTAAGGACATCGCCTACCA	CCTGGCTTTCTCGTCACTCTCA
ADIPO	CAGGCCGTGATGGCAGAGATG	GGTTTCACCGATGTCTCCCTTAG
LPL	CTGCTGGCATTGCAGGAAGTCT	CATCAGGAGAAAGACGACTCGG
COMP	GGAGATGCTTGTGACAGCGATC	TGAGTCCTCCTGGGCACTGTTA
ACAN	TGGTGATGATCTGGCACGAG	CGTTTGTAGGTGGTGGCTGTG
IL6	GCCACTCACCTCTTCAGAAC	GCCTCTTTGCTGCTTTCACAC
INF‐γ	CCAAGTGATGGCTGAACTGTCG	GCAGGCAGGACAACCATTACTG
TNF‐β	CACCTCCCTGAACCATCCCTGAT	CTCCATGTGCCTGCTCTTCCTCT
Ki‐67	CGACCCTACAGAGTGCTCAACAA	CTTGTCAACTGCGGTTGCTCCTT
PCNA	CCACTCTCTTCAACGGTGACACT	CATCCTCGATCTTGGGAGCCAA

### 2.7 Differentiation assays

For osteogenic differentiation, cells were induced by growth in classic MSCs medium supplemented with 10 mmol/L β‐glycerophosphate disodium salt hydrate (Sigma, Cat# G9422), 50 μmol/L l‐ascorbic acid (Med Chem Express, Cat# HYB0166) and 100 nmol/L dexamethasone (Sigma, Cat# D4902). For adipogenic differentiation, the induction medium was classic MSCs medium supplemented with 1 μmol/L dexamethasone (Sigma, Cat# D4902), 0.5 mmol/L IBMX (Sigma, Cat# I7018), 200 μmol/L indometacin (Sigma, Cat# I7378) and 10 μmol/L insulin (Meilunbio, CAT# 11061680). For chrondrogenic differentiation, the induction medium was classic MSCs medium supplemented with 50 μmol/L l‐ascorbic acid (Med Chem Express, Cat# HYB0166), 1 μmol/L dexamethasone (Sigma, Cat# D4902), 1% insulin‐transferrin‐sodium selenite media supplement (ITS) (Gibco, Thermo Fisher Scientific, Cat# 41400045), 500 μmol/L sodium pyruvate solution (Sigma, Cat# S8636) and 10 μg/L TGF‐β1 (Novoprotein, Cat# CA59). After 21 days of differentiation, special stains were used for qualitative assay: alizarin red S (Solarbio, CAT# G1452) for osteocytes, oil red O (Solarbio, Cat# G1260) for adipocytes and toluidine blue O (Solarbio, Cat# G2543) for chondrocytes. UCMSCs were used as a positive control, and AFMSCs without differentiation were the negative control. RT‐PCR of specific genes was used as a quantitative assay of cell differentiation capability.

### 2.8 Immunosuppression of PHA‐stimulated PBMCs assays

For co‐culture, MSCs were counted, seeded and attached for 6 hours; then, they were pre‐treated for 3 hours with 10 µg/mL mitomycin C (Med Chem Express, Cat# HY13316) to inhibit cell proliferation. PBMCs from healthy donors were labelled with the fluorescent dye CFSE (Invitrogen, Cat# C34554) and were then seeded at a ratio of 1:5 (MSCs versus PBMCs) into the wells where MSCs were attached in. The induced medium was RPMI‐1640 (CORNING, Cat# 10040CVR) supplemented with PHA (Dahui Biotechnology, Cat# 20190704), 30% FBS (Gibco) and 100 U/mL penicillin and 100 μg/mL streptomycin (Gibco). After 72 hours of co‐culture, suspension cells were collected for two assays. One was flow cytometry to determine the CFSE fluorescence ratio, and the other was RT‐PCR for the measurement of mRNA expression.

### 2.9 Statistical analysis

All experiments were repeated at least three times for statistical analysis. For independent results, data were expressed in a scatter plot. For separate samples, quantitative results were expressed as the means ± SD. Differences between two groups were statistically analysed by unpaired Student's *t* tests. Multigroup comparisons were analysed by Tukey's test, and ANOVA was used to compare the groups' differences in a time‐dependent manner. A difference was considered significant if the *P*‐value was < .05.

## 3 RESULTS

### 3.1 AFMSCs derived from different culture media

**Figure 2:**
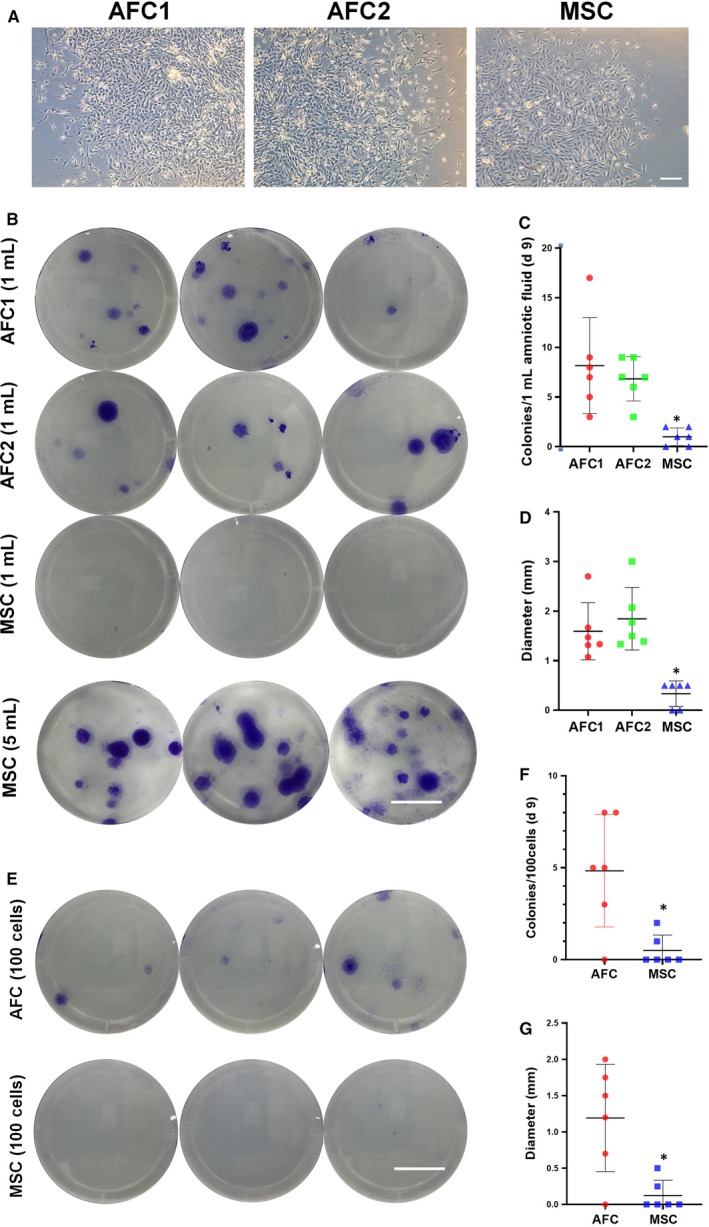
Primary culture of AFMSCs uses commercial AFC and classic MSC culture medium. Cell colonies were formed by AFCs incubated in commercial AFC1, AFC2 and classic MSCs culture medium on day 7‐9 (A), bar = 100 μm. Crystal violet staining of three independent primary colonies in different culture medium (B) (Bar = 1 cm), which seeded 1 mL or 5 mL amniotic fluid. Statistical analysis of primary colony numbers (C) and average diameter (D) after cells of 1 mL amniotic fluid seeded after 9 d primary culture (n = 5, *P < .05 versus AFC1 group). Crystal violet staining of three independent passaged colonies in different culture medium (E) (Bar = 1 cm), which seeded 100 AFCs. Statistical analysis of colony numbers (C) and average diameter (D) (n = 6, *P < .05 versus AFCs group). AFC was indicated AFMSCs cultured by AFC1 media

Cell pellets from amniotic fluid were seeded at a clonal density and incubated in two commercial AFC medium and classic MSC medium, and colonies appeared on day 7. Then, we discarded the floating cells and fed the attached cells with a fresh medium for the remaining two days of culture. Colony morphology was observed using bright field microscopy (Figure 2A), and crystal violet staining was used to determine the number and diameter of colonies (Figure 2B). There was no difference in AFC1 and AFC2 group, while the number of colonies was significantly lower (Figure 2C) and the diameter was significantly smaller in the MSC group compared with the AFC groups (Figure 2D). Furthermore, we investigated the colonies formation ability of passaged AFCs incubated in AFC1 and MSC medium. Hundred cells were seeded into 10 cm^2^ culture plate and stained by crystal violet after 9 days culturing (Figure 2E). There were significant decreases of colonies' number (Figure 2F) and diameter (Figure 2G) in MSC groups compared with AFC groups.

### 3.2 AFMSCs grown with different culture methods share cell morphology and most cell surface markers

**Figure 3:**
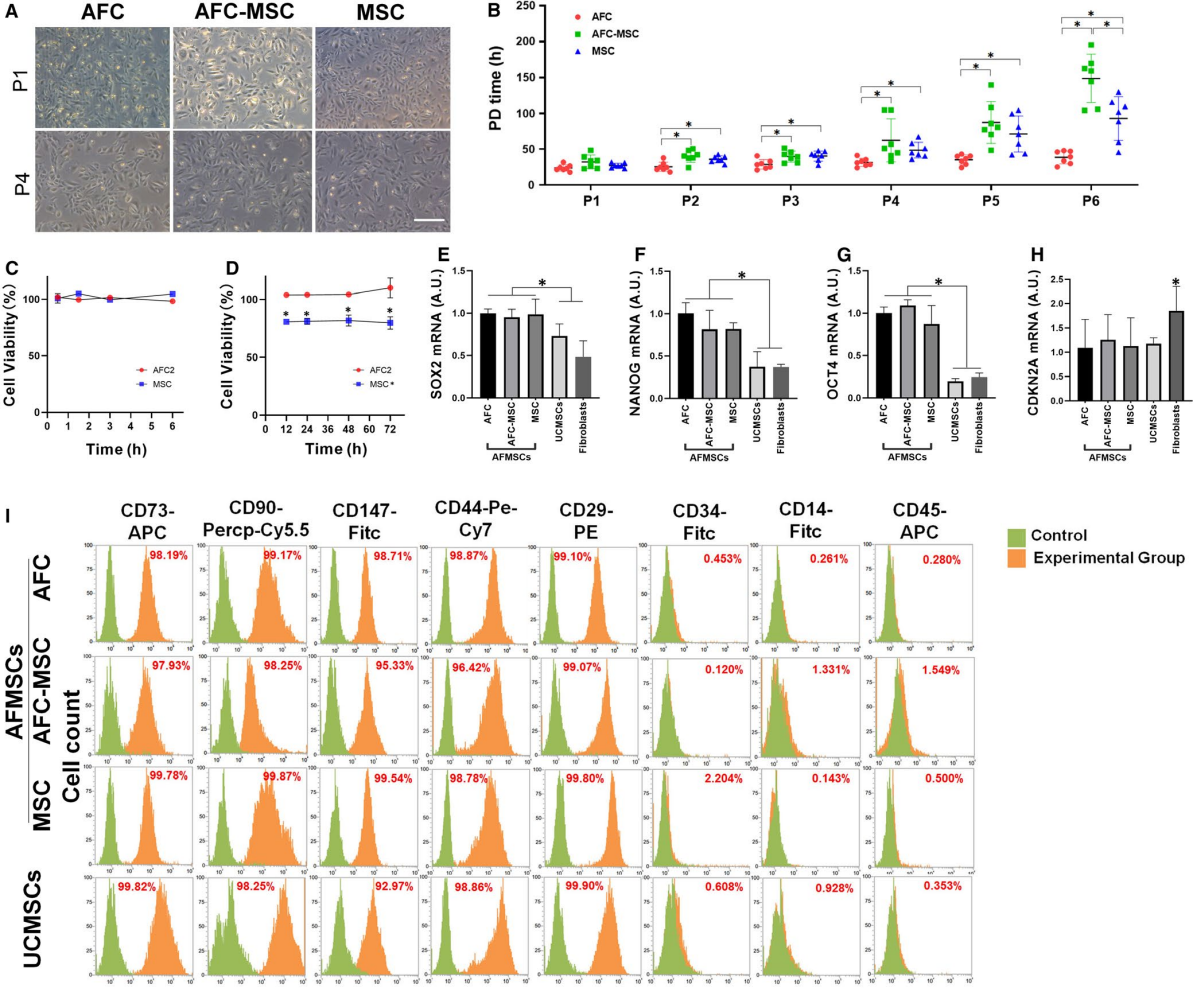
Subculture of AFMSCs in different culture schemes. Cell morphology of AFMSCs in different schemes on passage 1 and passage 4 (A), bar = 100 μm. Cell population double time (B) of AFMSCs was assayed from passage 1 to passage 6 (n = 7, *P < .05). Cell viability was assayed in time manners to assess cell attachment (C) and cell proliferation (D) compared AFC2 and MSC to AFC1 group (n = 3, *P < .05 versus AFC1 group, representatively). Cell viability of AFC1 was regarded as a reference, but was not given in the chart. The trend lines in C and D were indicated the cell viability change compared to reference AFC1. AFMSCs of passage 4 were assayed mRNA expression of SOX2 (E), NANOG (F), OCT4 (G) and CDKN2A (H) (n = 3, *P < .05). Flow cytometry analysis was used for AFMSCs and UCMSCs' surface markers expression. Cells were positive of CD73, CD90, CD147, CD44 and CD29, but negative of CD34, CD14 and CD45 (I). AFC was indicated AFMSCs cultured by AFC1 media

AFMSCs were subcultured in commercial AFC medium and classic MSC medium. There was no significant difference in cell morphology (Figure 3A). Cell proliferation was expressed by a population doubling (PD) time assay from passage 1 to passage 6. AFCs showed a significantly shorter PD time from passage 2 to passage 6; in long‐term culture (passage 6), MSC derived AFMSCs showed a shorter PD time than AFC‐MSC groups (Figure 3B). To determine the effect of AFC1, AFC2 and MSC medium on cell attachment and proliferation, we seeded the same quantity of AFMSCs and assayed cell viability at different times. For each time point, cell viability of AFC2 and MSC was statistical analysis compared with AFC1; AFC1 was regarded as a reference. In 6 hours, cell viability was used to express cell attachment capability, and there were no differences between the three groups (Figure 3C). In 72 hours, cell viability was used to express cell proliferation capability and the result showed there was still no difference between AFC1 and AFC2 groups, and significantly lower in MSC groups compare with AFC groups (Figure 3D). As enough cells were produced with 4 passages, the following study used cells of passage 4 or passage 5. The mRNA expression of pluripotent markers in AFMSCs was significantly higher than it was in UCMSCs and fibroblasts. There was no significant change in the ageing‐associated marker CDKN2A between AFMSCs and UCMSCs, while the expression in fibroblasts was higher (Figure 3E‐H). AFMSCs shared the same surface markers as UCMSCs, which consisting of CD73, CD90, CD147, CD44 and CD29, but they were negative for CD34, CD14 and CD45 (Figure 3I).

### 3.3 The culture scheme causes AFMSCs to differ in cell morphology, CD105 expression and tissue‐specific markers

**Figure 4:**
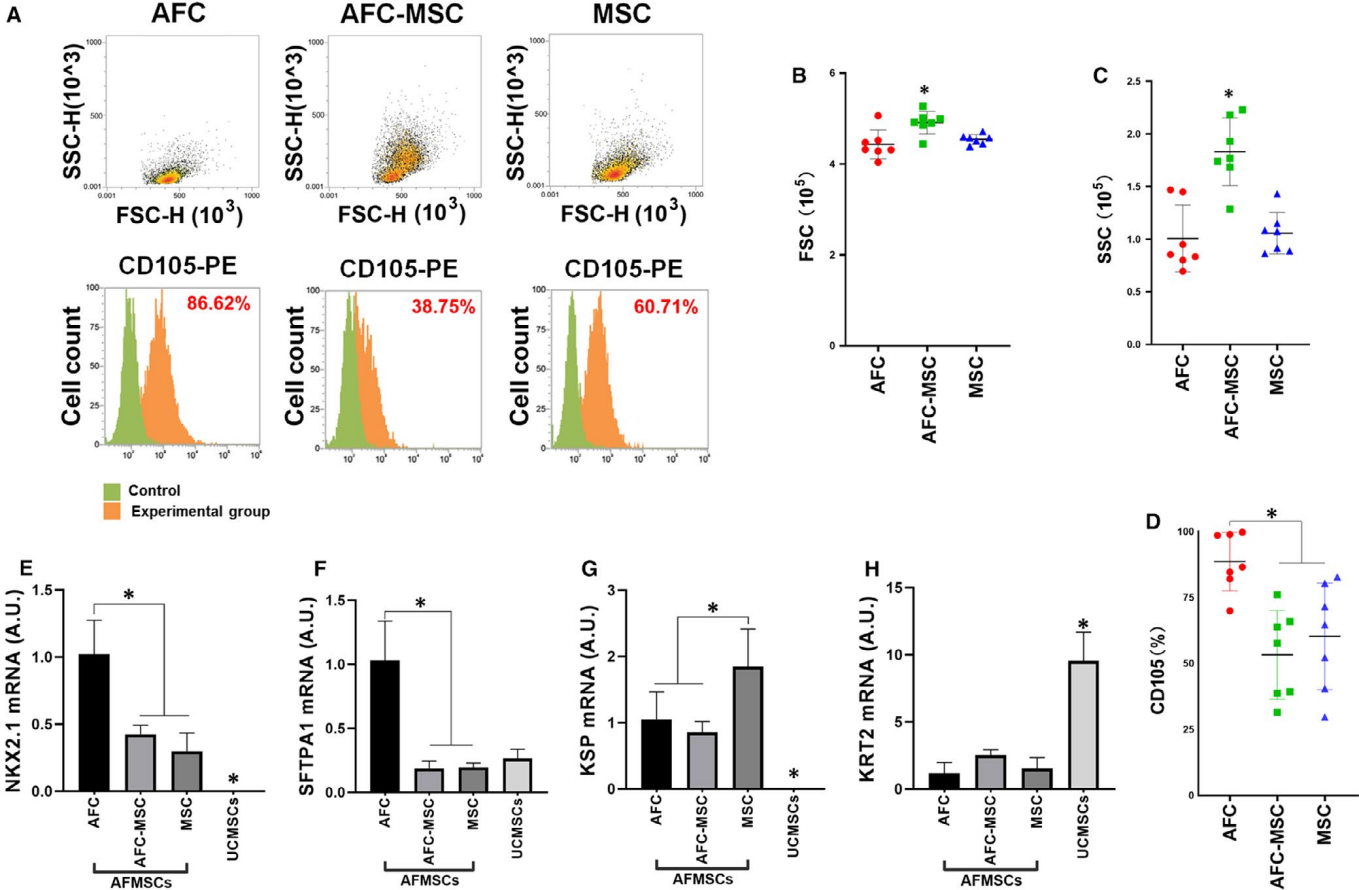
Different culture schemes leading difference of cell size, positive level of CD105 and tissue‐specific markers. After different culture schemes, the difference of passage 4 AFMSCs was assayed. Cell size and expression of CD105 were detected by flow cytometry analysis (A). Statistical analysis of FSC (B), CSC (C) and CD105 expression (D) was assayed in different groups. mRNA expression of tissue‐specific markers was assayed by RT‐PCR, NKX2.1 (E) and SFTPA1 (F) were specific for lung, KSP (G) was for kidney, and KRT2 (H) was for skin (n = 3, *P < .05; * above UCMSCs group was significant difference compare with AFMSCs). AFC was indicated AFMSCs cultured by AFC1 media

There were some differences in AFMSCs produced by the different culture methods. Based on flow cytometry analysis (Figure 4A), FSCs and SSCs were significantly higher than AFC and MSC group (Figure 4B,C). The expression of CD105 in AFC was significantly higher than that in AFC‐MSC and MSC groups (Figure 4D). We used RT‐PCR to test the mRNA expression of specific lung, kidney and skin tissue markers (Figure 4E‐H). AFMSCs expressed significantly higher levels of NKX2.1 and KSP but expressed lower levels of KRT2 compared with UCMSCs. For the AFMSCs groups in different culture methods, AFCs expressed significantly higher levels of lung markers (NKX2.1 and SFTPA1), while MSCs expressed significantly higher levels of a kidney marker (KSP).

### 3.4 AFMSCs acquire enhanced differentiation capability compared with UCMSCs

**Figure 5:**
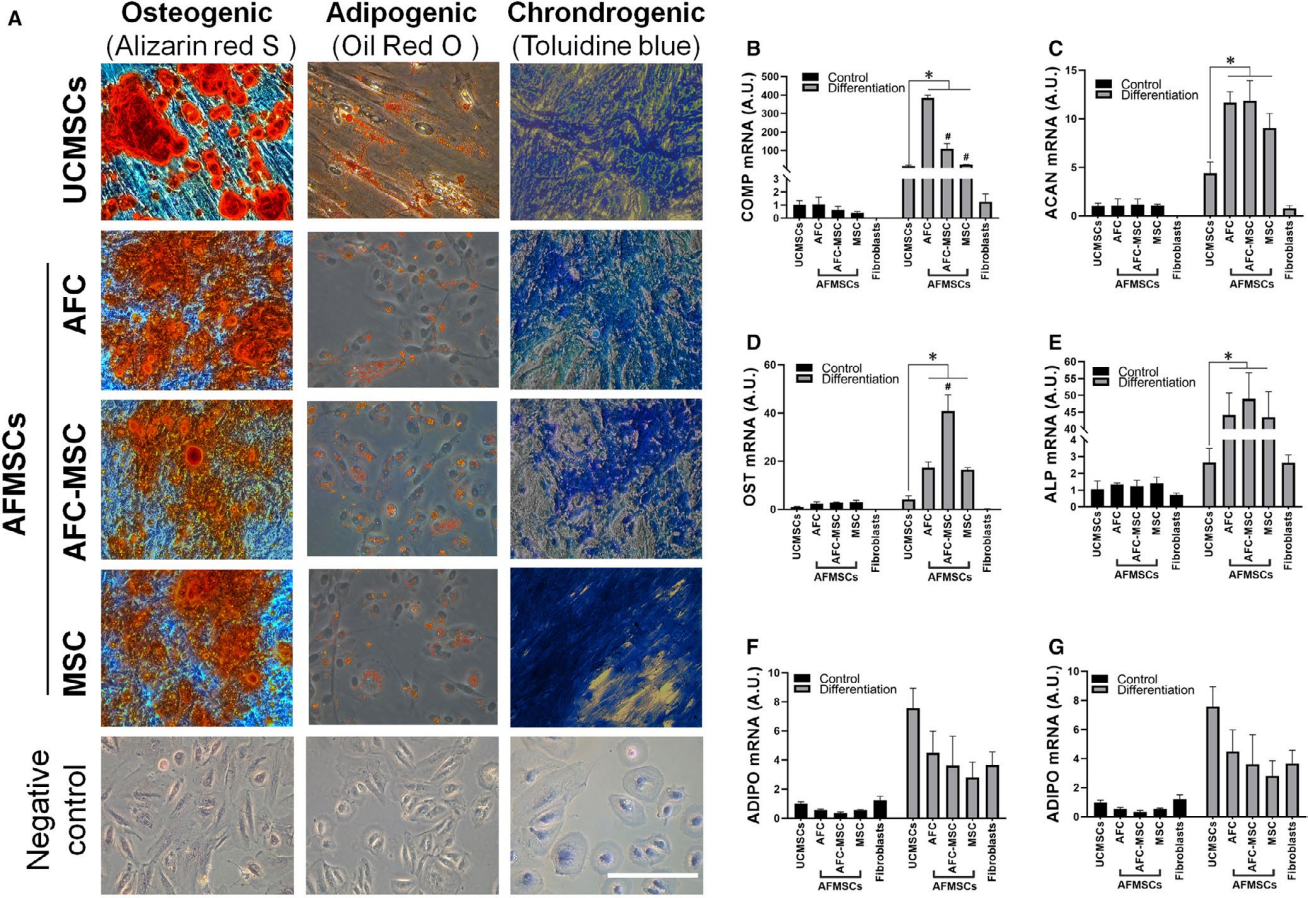
Differentiation capability of AFMSCs. Cell special dyeing of AFMSCs and UCMSCs differentiation to osteocytes, adipocytes and chondrocytes (A). RT‐PCR was conducted to assay mRNA expression of tissue‐specific markers, OST (B) and ALP (C) were for osteogenic, ADIPO (D) and LPL (E) were for adipogenic, and COMP (F) and ACAN (G) were for chrondrogenic (n = 3, *P < .05; #*P* < .05 versus to AFCs group). AFC was indicated AFMSCs cultured by AFC1 media

AFMSCs shared the capability of differentiation into osteocytes, adipocytes and chondrocytes with UCMSCs, as indicated by the positive cells (stained with tissue‐specific dye) after induction (Figure 5A) and the expression of tissue‐specific mRNA after induced differentiation. With the aim of quantitative analysis of variations in differentiation capability, two tissue‐specific markers were assayed in each committed differentiation. For adipogenesis, no significant difference was found (Figure 5D,E). For osteocytes, and chondrocytes, the differentiated AFMSCs expressed significantly higher levels of OST, ALP, COMP and ACAN compared with UCMSCs. These data suggested that the ability to differentiate into osteocytes, and chondrocytes were enhanced, but the same trend was not observed in AFMSCs (Figure 5B,C,F,G).

### 3.5 AFMSCs were exhibited enhanced immunosuppression of PHA‐activated PBMCs compared with UCMSCs

**Figure 6:**
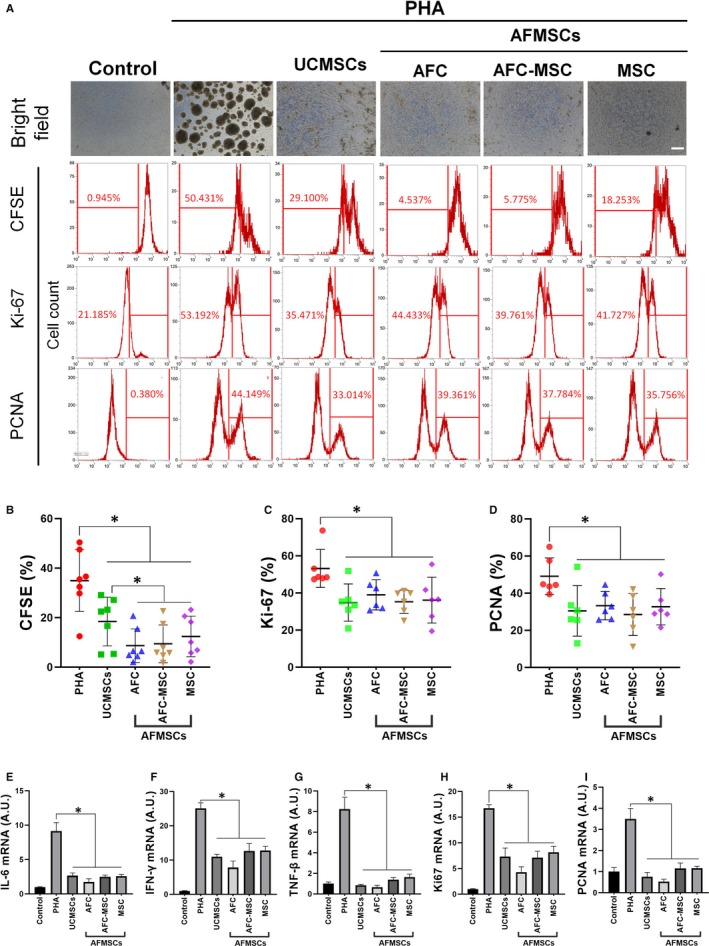
AFMSCs immunosuppress PHA‐stimulated PBMCs. Bright field observation and flow cytometry analysis of CFSE, Ki67 and PCNA were used for qualitative and quantitative analysis of PHA‐stimulated PBMCs and UCMSCs and AFMSCs immunosuppression effect (A), bar = 100 μm. Statistical analysis of CFSE (B), Ki67 (C) and PCNA (D) positive rate in different groups (n = 7 for CFSE, n = 6 for Ki67 and PCNA, *P < .05). Specific genes expression of mRNA was assayed, IL‐6 (E), IFN‐γ (F) and TNF‐β (G) were for immuno‐activity, and Ki67 (H) and PCNA (I) were for proliferation (n = 3, *P < .05). AFC was indicated AFMSCs cultured by AFC1 media

AFMSCs share same immunosuppression effect with UCMSCs as inhibit PHA induced human PBMCs aggregation, decrease the percentage of CFSE‐negative, Ki67‐ and PCNA‐positive cells (Figure 6A). For statistical analysis, AFMSCs groups were significantly lower ratio of CFSE‐negative (Figure 6B), Ki67‐ (Figure 6C) and PCNA (Figure 6D)‐positive PBMCs compared with UCMSCs groups. AFMSCs and UCMSCs were sharing similar immunosuppression effect of inhibit PHA induced mRNA expression of immune‐cytokine and cell proliferation markers, but there were no significant in compare of AFMSCS and UCMSCs or AFMSCs in different culture methods (Figure 6E‐I).

## 4 DISCUSSION

The concept of MSCs is used to describe cells with following criteria, including adherence to plastic; expression of cell surface markers CD105, CD90 and CD73; lack of haematopoietic and endothelial markers; and ability to differentiate into cartilage, bone and fat by chemical induction.^3^ MSCs could be derived and propagated from most organs and tissues,^19^ and the basic criteria for defining MSCs did reflect them in vivo performance.^20^ Therefore, seeking a reliable cell source and clarifying the relationship between their characteristics and their biological functions is important in MSC studies. Our work was focused on MSCs derived from amniotic fluid, and we assessed the production process and associated characteristics that would broaden the use of MSCs and illustrated the relationship between AFMSC production and their biological functions.

Prenatal diagnosis by cytogenetic analysis of cultured AFCs has been used in the clinic for decades.^21^ There are commercial protocols for AFC culture that include the addition of cytokines and pregnancy hormones to the culture medium to optimize the number of primary colonies and increase cell proliferation; most of the AFMSC studies used biological additives, such as Chang B and Chang C,^22^ which was different with the culture system of MSC studies.^10^ For clinical usage, MSCs were derived and cultured in a serum‐free system,^23^ but in most studies, MSCs were isolated through attachment to plastic agents and were cultured in DMEM with foetal bovine serum (FBS) or other additives based on the specific method.^10^ Here, we used commercial AFCs and classic MSCs culture medium to isolate and culture AFMSCs and systematically evaluate the difference in primary colony formation, cell doubling time, cell surface marker expression, mesoderm cell differentiation capability and immunosuppression of PHA‐activated PBMCs. AFMSCs were cultured by three different culture media, and the cells exhibited characteristic differentiation into typical somatic mesoderm tissues and had immunosuppression activity. The commercial AFC medium showed some advantages in primary colony formation and cell proliferation. However, comprehensive consideration of economic cost and high‐quality cell products makes the use of AFCs a good choice for primary culture and MSC medium a good choice for subculture.

There were two generic methods used to isolate MSCs: one was sorting based on the expression of specific markers (such as CD146 from bone marrow^24^ and CD117 from amniotic fluid^25^), and the other was through direct adherent culture. MSCs could form colonies (which were regarded as colony‐forming units‐fibroblastic [CFU‐Fs]) at clonal density in primary culture of bone marrow or amniotic fluid because they were derived from single cells with enormous proliferative capacity. Primary cultures were isolated by limiting the growth to clonogenic cells, which was an effective way to acquire antigenic homogeneity amongst cells for immunoselection.^26^ MSCs are non‐immortalized cells, and subculture encompasses a progressive loss of proliferation ability and a declining differentiation potential^27^; replicative senescence occurs after 20 to 40 rounds of division.^9^ We isolated clonogenic cells by seeding cells at a clonal density to obtain relatively homogeneous MSCs during AFMSC primary culture. AFMSCs exhibited a stable PD time when expanded in commercial AFC medium, while classic MSC medium led to a significant change in PD time, cell morphology, mRNA expression of tissue‐specific genes and CD105‐positive level; all cultures produced one billion cells in 5 passages.

As one kind of MSC, AFMSCs derived from amniotic fluid show some advantages over MSCs from other sources. For biological sampling, amniocentesis was safer and less harmful to the patient than bone marrow puncture. Second, it was easy to isolate primary cells from amniotic fluid. AFCs were separated from their liquid, and the primary culture of AFMSCs was used to collect the cell pellets and seed them into certain media. MSCs derived from umbilical cord and placental required the isolation of cells from tissue pieces. Finally, AFMSCs exhibited some biological advantage in cytotherapy potential, such as efficiency in dealing with senescence stress,^28^ yielding exosomes,^29^ having a widespread histologic origin^30^ and having potent immunomodulatory effect on T cells.^31^ In this study, we established an AFMSC culture method and systematically evaluated the differences between AFMSCs, UCMSCs and fibroblasts. AFMSCs showed high expression of pluripotent‐associated genes, enhanced capability of osteogenic and chrondrogenic differentiation and immunosuppression of PHA‐activated PBMCs.

CD105 is a homodimeric transmembrane protein that is a component of the transforming growth factor beta receptor (TGFBR) complex, which is encoded by endoglin and expressed in most organs. One criterion for identifying MSCs is expression of CD105, but there were different reports on the reliability of this biomarker. First, MSCs derived from different cell sources did not express CD105, BMMSCs were composed almost exclusively of CD105‐positive cells,^32^ and AFMSCs were partly positive for CD105.^28^ In our previous study, the number of CD105‐positive cells in AFCs was significant increasing in long‐term culture,^21^ which consist with this study and indicate that AFCs cells are heterogeneous, and AFCs culture is a process of biological select CD105‐positive cells or induce CD105 expression. Thus, the biological function of CD105 in MSCs is debatable, and its expression does not predict chondrogenic potential in BMMSCs,^32^ while it promotes chondrogenesis of synovium‐derived MSCs.^33^ In this study, we demonstrated that most of the CD markers of AFMSCs cultured in different media were stable except CD105, this phenomenon was not associated with cell differentiation capability or an immunological effect.

## 5 CONCLUSIONS

In summary, we demonstrated that AFMSCs could be isolated and cultured on commercial AFCs medium and classic MSCs culture medium, which exhibited more powerful osteogenic and chrondrogenic differentiation potential and immunosuppression effects of PHA‐activated PBMCs. AFMSCs are excellent MSCs for cytotherapy, and they are generated with little trauma for patients and provide cell sources for the lung and kidney. Expression of CD105, as a usual surface marker of MSCs, changed in AFMSCs grown with different protocols, but this was not associated with AFMSC biological function. Additional studies are needed to determine whether AFMSCs could be a cytotherapy agent that participates in the treatment of pathology and disease.

## CONFLICT OF INTEREST

The authors declare no conflict of interest.

## AUTHOR CONTRIBUTIONS

DW and XS carried out study design. DW, NL, YX, BS and SK performed the experiments. DW, NL and YX did statistical analysis. DW and NL wrote the paper. All authors read and approved the final manuscript.

## Data Availability

The data used to support the findings of this study are included within the article.
